# Bassoon inhibits proteasome activity via interaction with PSMB4

**DOI:** 10.1007/s00018-020-03590-z

**Published:** 2020-07-10

**Authors:** Carolina Montenegro-Venegas, Sandra Fienko, Daniela Anni, Eneko Pina-Fernández, Renato Frischknecht, Anna Fejtova

**Affiliations:** 1 Department of Neurochemistry and Molecular Biology, Leibniz Institute for Neurobiology, Magdeburg, Germany; 2 Molecular Psychiatry, Department of Psychiatry and Psychotherapy, University Hospital, Friedrich-Alexander-University Erlangen-Nürnberg, Erlangen, Germany; 3 RG Presynaptic Plasticity, Leibniz Institute for Neurobiology, Magdeburg, Germany; 4 Department of Biology, Animal Physiology, Friedrich-Alexander University of Erlangen- Nürnberg, Erlangen, Germany; 5 Present Address: Institute for Pharmacology and Toxicology, Otto-von-Guericke University and Center for Behavior Brain Sciences (CBBS), Magdeburg, Germany; 6 Present Address: Huntington’s Disease Centre, Department of Neurodegenerative Disease, UK Dementia Research Institute at UCL, Queen Square Institute of Neurology, University College London, London, WC1N 3BG UK

**Keywords:** Protein degradation, Ubiquitin–proteasome system, Synapse, Cytomatrix at the active zone, Proteostasis

## Abstract

**Abstract:**

Proteasomes are protein complexes that mediate controlled degradation of damaged or unneeded cellular proteins. In neurons, proteasome regulates synaptic function and its dysfunction has been linked to neurodegeneration and neuronal cell death. However, endogenous mechanisms controlling proteasomal activity are insufficiently understood. Here, we describe a novel interaction between presynaptic scaffolding protein bassoon and PSMB4, a β subunit of the 20S core proteasome. Expression of bassoon fragments that interact with PSMB4 in cell lines or in primary neurons attenuates all endopeptidase activities of cellular proteasome and induces accumulation of several classes of ubiquitinated and non-ubiquitinated substrates of the proteasome. Importantly, these effects are distinct from the previously reported impact of bassoon on ubiquitination and autophagy and might rely on a steric interference with the assembly of the 20S proteasome core. In line with a negative regulatory role of bassoon on endogenous proteasome we found increased proteasomal activity in the synaptic fractions prepared from brains of bassoon knock-out mice. Finally, increased activity of proteasome and lower expression levels of synaptic substrates of proteasome could be largely normalized upon expression of PSMB4-interacting fragments of bassoon in neurons derived from bassoon deficient mice. Collectively, we propose that bassoon interacts directly with proteasome to control its activity at presynapse and thereby it contributes to a compartment-specific regulation of neuronal protein homeostasis. These findings provide a mechanistic explanation for the recently described link of bassoon to human diseases associated with pathological protein aggregation.

**Graphic Abstract:**

Presynaptic cytomatrix protein bassoon (Bsn) interacts with PSMB4, the β7 subunit of 20S core proteasome, via three independent interaction interfaces. Bsn inhibits proteasomal proteolytic activity and degradation of different classes of proteasomal substrates presumably due to steric interference with the assembly of 20S core of proteasome. Upon Bsn deletion in neurons, presynaptic substrates of the proteasome are depleted, which can be reversed upon expression of PSMB4-interacting interfaces of Bsn. Taken together, bsn controls the degree of proteasome degradation within the presynaptic compartment and thus, contributes to the regulation of synaptic proteome
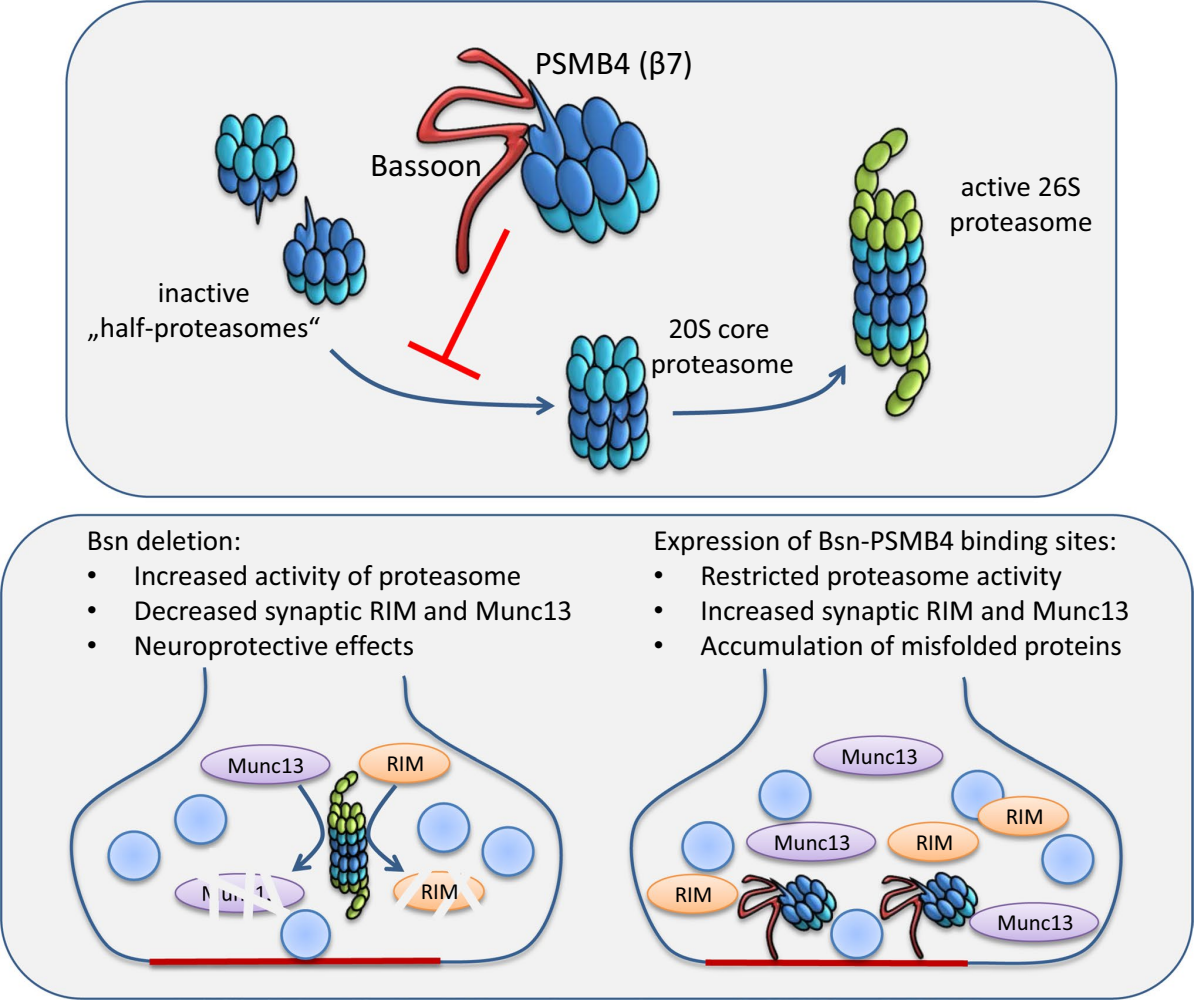

**Electronic supplementary material:**

The online version of this article (10.1007/s00018-020-03590-z) contains supplementary material, which is available to authorized users.

## Introduction

Neurons are long-living cells with a complex morphology which imposes challenges and constraints on cellular proteostasis network [47]. To ensure subcellular functional diversity, dendritic, axonal or synaptic proteomes are shaped by compartment-specific protein supply and degradation [5, 6, 53]. The ubiquitin (Ub)-proteasome system (UPS) potentially contributes to this, since it plays a major role in the regulation of cellular protein turnover by mediating protein ubiquitination and subsequent degradation. In line with that, a number of presynaptic proteins have been identified to be ubiquitinated or regulated by the proteasome [16, 34].

Despite a growing list of synaptic UPS targets, our understanding of mechanisms controlling its activity in neurons remains rudimental. It has been proposed that substrate selectivity of Ub-conjugating enzymes plays a key role in determining UPS specificity [31, 52]. Thus, regulation of the activity of Ub-ligases might be one of the mechanisms that selectively target a subset of cellular proteins for degradation by the UPS. However, increasing number of studies suggests a proteasome-independent role of dynamic ubiquitination in synaptic signaling and thus additional mechanisms should exist that regulate assembly, subcellular localization or processivity of the proteasome [30, 39]. Recently, a new proteasome species located to neuronal membrane has been identified. This specialized proteasome lacks the 19S regulatory particle necessary for Ub-recognition of target proteins and is responsible for activity-dependent production of secretory peptides with signaling function [37]. Thus, highly specialized roles for synaptic UPS have emerged that likely rely on synapse-specific regulations.

Presynaptic scaffolding proteins bassoon (Bsn) and piccolo (Pclo), which are vertebrate-specific organizers of the cytomatrix at the active zone, have been repeatedly linked to the regulation of neuronal proteostasis [15]. These proteins interact with and negatively regulate the activity of E3 Ub-ligase seven in absentia homologue 1 (Siah1) [46]. Siah1 acts in concert with adenomatous polyposis coli‐based SCF E3 ligase complex to ubiquitinate several neuronal proteins including SV protein synaptophysin (Syp) [48]. Deletion of both Bsn and Pclo increases ubiquitination of SV proteins and leads to progressive neuronal degeneration, which is likely driven by destabilization of SV cluster at presynaptic sites and their degradation by proteasomal and endo-lysosomal pathways [46]. Recently, Bsn has been reported to interact with autophagy-associated E3-like ligase Atg5 and suppress its activity, controlling thereby presynaptic autophagy [20, 36]. Thus, Bsn regulates ubiquitination and degradation of synaptic proteins via proteasomal, endo- lysosomal and autophagocytic pathways, which supports recently emerging view of the large functional interplay among these pathways in neurons [47]. Interestingly, two recent clinical reports further support a key role of Bsn in the regulation of neuronal proteostasis. In the first study, homozygous missense mutations in BSN has been linked with familiar and sporadic progressive supranuclear palsy (PSP)-like syndrome associated with tauopathy [49]. In the second, Bsn has been identified as a constituent of toxic accumulates in the somata of motoneurons in mice and patients with multiple sclerosis [41]. Strikingly, overexpression of Bsn was neurotoxic, while genetic disruption of Bsn protected mice from inflammation-induced neuroaxonal injury. A similar protective effect was observed following pharmacological proteasome activation indicating a functional link between Bsn and the proteasome [41].

In this work, we describe a novel interaction between Bsn and PSMB4, the β7 structural subunit of the 20S core proteasome. Expression of PSMB4-interacting Bsn fragments inhibited multiple endopeptidase activities of endogenous proteasomes and induced strong accumulation of soluble and misfolded ubiquitination-dependent as well as ubiquitination-independent proteasome substrates. These effects are distinct and independent from the previously described regulation of autophagy or ubiquitination-dependent proteasomal degradation linked to interactions of Bsn with Atg5 and Siah1, respectively, and might rely on a steric interference with the assembly of the 20S core proteasome. In line with a negative regulatory role of Bsn on the endogenous synaptic proteasome, we measured increased proteasomal activity in the synaptosomes prepared from brains of Bsn deficient mice and synaptic levels of known proteasome substrate proteins were decreased in primary neurons derived from these animals. Finally, expression of Bsn fragments containing PSMB4-interaction sites on Bsn deficient background normalized increased activity of proteasome and lower expression levels of presynaptic proteasome substrates. Thus, we propose that Bsn directly interacts with proteasome to control its activity at presynapse and thereby it contributes to a compartment-specific control of neuronal protein homeostasis.

## Results

### Bsn interacts with PSMB4 through three independent interaction interfaces

**Fig. 1:**
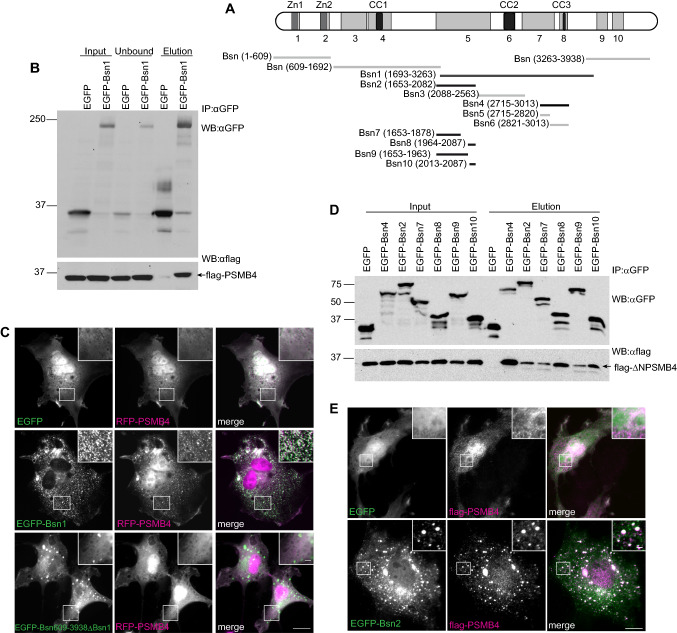
Characterization of the PSMB4-interaction interface on Bsn. **a** The schema depicts the localization of fragments used in this study on Bsn. Black bars denote fragments that associate with PSMB4, whereas grey are non-interacting fragments. Abbreviations: Zn1/2, zinc fingers; CC1-3, coiled-coil regions 1 to 3. Numbers of respective amino acid residues of rat Bsn are given in brackets. **b** Flag-PSMB4 is detectable in all lysates prepared from transfected HEK293T (Input) but co-immunoprecipitates only with EGFP-Bsn1, and not EGFP as shown by immunoblotting of elution fractions. **c** RFP-PSMB4 (aa 32–263) is recruited to clusters formed by EGFP-Bsn1 in COS7 cells. Deletion of the entire binding segment (aa 1692–3263) interferes with co-recruitment of RFP-PSMB4. **d** Flag-ΔNPSMB4 is detected in all tested cell lysates but co-precipitates only with EGFP-Bsn4, EGFP-Bsn7 and EGFP-Bsn10 but not with EGFP. **e** Recruitment assay in COS7 cells reveals that flag-PSMB4 (aa 32–263) is recruited to EGFP-Bsn2-containing clusters. Co-IP experiments were conducted three to four times on independent HEK293T cell cultures. The insets show magnified regions in squares. Scale bars represent 10 μm in overview and 2 μm in inset

**Fig. 2:**
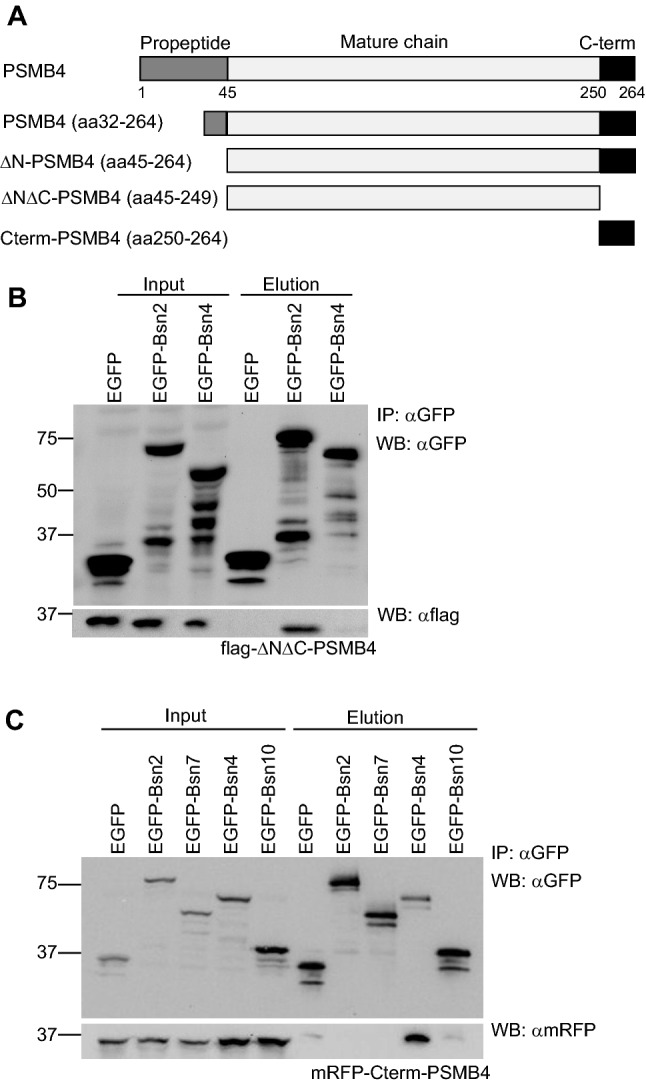
Bsn binds different domains of PSMB4 protein. **a** Schematic diagram depicting the structure of PSMB4 and the fragments used in Co-IP experiments. **b** GFP antibodies co-precipitate PSMB4 deprived of N- and C-terminus (flag-ΔNΔC-PSMB4) when co-expressed with EGP-Bsn2, but not with EGFP-Bsn4 or EGFP alone. **c** EGFP-Bsn4, but not Bsn2 or EGFP, bind C-terminal domain of PSMB4 (mRFP-CtermPSMB4). Co-IP experiments were performed three to four times on independent HEK293T cell cultures. All transfected constructs are detectable in the respective input fractions

Proteasome subunit beta type 4 (PSMB4) was identified to interact with Bsn in a yeast two-hybrid (Y2H) screen with the bait fragment Bsn1 spanning amino acids (aa) 1692–3263 of rat Bsn (Fig. 1a). Bsn fragments covering aa 1–609, aa 609–1692 and aa 3263–3938 did not show any interaction with PSMB4 in this assay. PSMB4, also known as β7, is one of seven subunits of β-ring of the 20S core proteasome [4, 12]. PSMB4 is expressed with a 45 aa-long N-terminal propeptide sequence, which is processed during 20S particle assembly [19]. In the initial screen, two independent prey clones were isolated, both bearing the sequence of N-terminally truncated PSMB4 starting with aa 86, suggesting that interaction interface maps to the mature peptide of PSMB4. To confirm the results obtained in the Y2H screen, we performed co-immunoprecipitation (co-IP) experiments from lysates of HEK293T cells expressing flag-PSMB4 (aa 32–264, Fig. 2a) together with either EGFP-Bsn1 or EGFP using GFP antibodies. Flag-PSMB4 co-precipitated with EGFP-Bsn1 but not with EGFP confirming the specific association of the mature chain of PSMB4 with Bsn1 in mammalian cells (Fig. 1b). To further support these results, we performed co-clustering studies in COS7 cells expressing mRFP-PSMB4 and either EGFP, EGFP-Bsn1 or EGFP-Bsn609–3938(Δ1692–3263) construct containing N-terminally truncated Bsn lacking the entire Bsn1 sequence. mRFP-PSMB4 showed diffuse cytoplasmic localization when co-expressed with EGFP, however, it was consistently recruited to the cytoplasmic clusters formed by EGFP-Bsn1 (Fig. 1c). In contrast, EGFP-Bsn609–3938(Δ1692–3263) that still formed punctate clusters in the cytoplasm, failed to co-recruit mRFP-PSMB4, indicating that Bsn1 sequence mediates interaction with PSMB4 in the cellular context (Fig. 1c). To further narrow down the PSMB4-binding interface on Bsn sequence, we generated shorter fragments of Bsn1 and tested them for binding to PSMB4 in a series of co-IP experiments. For these experiments, we used flag-ΔNPSMB4 (aa 46–264) constructs, which constitutes the mature peptide emerging upon cleavage of the 45 aa-long N-terminal propeptide [19]. This PSMB4 construct successfully co-precipitated with EGFP-fused fragments Bsn2, Bsn4, Bsn7, Bsn8, Bsn9 and Bsn10 but not with EGFP (Fig. 1d). Thus, three independent Bsn regions covered by fragments Bsn4 (aa 2715–3013), Bsn7 (aa 1653–1878), and Bsn10 (aa 2013–2087) could efficiently interact with PSMB4 (Fig. 1d), implying that multiple structural elements of Bsn contribute to PSMB4 binding. Moreover, EGFP-Bsn2 (comprising a sequence of Bsn7 and Bsn10) co-recruited flag-PSMB4 protein also in co-recruitment assay in COS7 cells (Fig. 1e). The shorter fragments Bsn5 and Bsn6 derived from Bsn4 did not bind PSMB4 (Fig. S1B, C). Interestingly, Bsn3 (aa 2088–2563) fragment that contains the CC2 domain and mediates interaction with Atg5 [36] showed no binding with PSMB4 (Fig. 1SA) indicating that Bsn associates with proteasome and autophagosome via independent sequence motives.

### Bsn binds two functionally distinct regions of PSMB4

In the next step, we mapped the interaction of individual Bsn fragments to the functional domains of PSMB4. The mature chain of PSMB4 consists of a globular domain with high homology to other subunits of the β-ring and a unique finger-like shaped C-terminal region (Fig. 2a). During the assembly of the 20S core proteasome, from two inactive half-proteasomes, this C-terminal region of PSMB4 intercalates between β subunits of the opposing half-proteasome [19]. This intercalation induces dimerization of two half-proteasomes and leads to the formation of the mature 20S particle [27, 38]. We generated two truncated constructs of flag-PSMB4: one lacking both N-terminal propeptide sequence as well as C-terminus (flag-ΔNΔC-PSMB4, aa 45–249) and the other covering only the C-terminal sequence (mRFP-Cterm-PSMB4, aa 250–264) and tested them for binding to Bsn fragments in Co-IP experiments (Fig. 2b, c). Flag-ΔNΔC-PSMB4 interacted with EGFP-Bsn2, but it failed to form a complex with EGFP-Bsn4 and control EGFP construct (Fig. 2b). In contrast, mRFP-Cterm-PSMB4 associated with EGFP-Bsn4, but did not bind EGFP-Bsn2, its shorter EGFP-tagged fragments Bsn7 and Bsn10 or control EGFP construct (Fig. 2c). These data imply that independent regions on Bsn interact with exclusive interfaces on PSMB4, raising the possibility for a functional segregation of these interactions. The binding of Bsn4 to the C-terminal region of PSMB4 implicated in the assembly of 20S core proteasome from half-proteasomes is intriguing since it might sterically hinder proteasome assembly.

### Bsn sequesters endogenous proteasome, is ubiquitinated, but not constitutively degraded

**Fig. 3:**
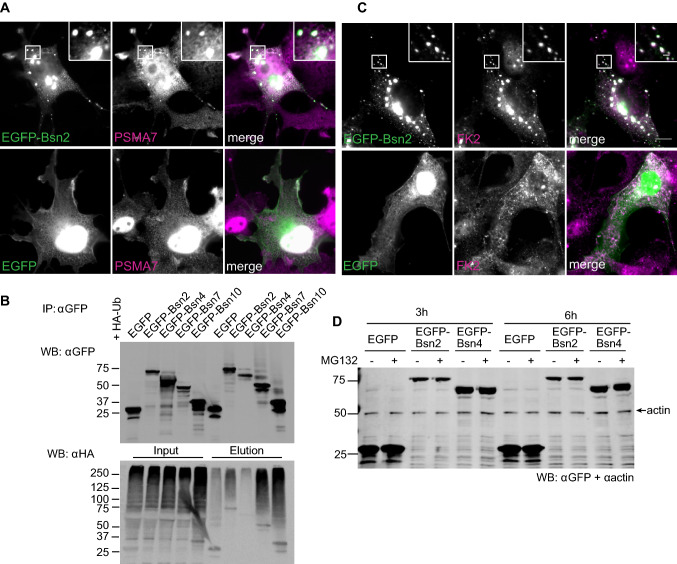
Bsn interacts with but it is not degraded by the endogenous proteasome. Endogenous proteasome subunit PSMA7 (**a**) and ubiquitin (**c**, stained with FK2 antibody) are recruited to EGFP-Bsn2-containing clusters in COS7 cells. Regions in squares are shown magnified in the inset in the upper left corner. Scale bars represent 10 μm in overview and 2 μm in inset. **b** EGFP-containing fragments were immunoprecipitated with specific GFP antibodies from HEK293T cells expressing EGFP, EGFP-Bsn2 or EGFP-Bsn4 together with HA-Ub. The expressed (Input) and co-precipitated (Elution) EGFP-fragments and therewith associated Ub moieties were detected by immunoblotting. Co-IP experiments were conducted three to four times on independent HEK293T cell cultures. Input samples confirm expression of all transfected constructs. **d** HEK293T cells were transfected with EGFP, EGFP-Bsn2 or EGFP-Bsn4 and treated with either 10 µM MG132 to block proteasome or DMSO for 3 or 6 h. Actin serves as a loading control. No differences in protein abundance were observed between conditions for all constructs

Next, we ask whether Bsn interacts with free PSMB4 or if it rather associates with this subunit in the context of the assembled proteasome. To this end, we expressed EGFP-Bsn2 or EGFP in COS7 cells and immuno-stained them for PSMA7, a proteasome subunit α4. Endogenous PSMA7 showed even distribution in the cytoplasm and in the nucleus of cells expressing EGFP, yet it was co-recruited to the cytoplasmic aggregates formed by EGFP-Bsn2 upon expression of this fragment (Fig. 3a) suggesting that this fragment recruits endogenous proteasome.

Interaction of Bsn2 and Bsn4 with PSMB4 might imply that these fragments are targeted for proteasomal degradation. An effective signal that directs proteins for degradation by the proteasome is their polyubiquitination [40]. To assess whether PSMB4-interacting Bsn fragments are polyubiquitinated, we overexpressed Bsn fragments together with HA-ubiquitin (HA-Ub), previously used to boost ubiquitination in HEK293T cells [24]. The association of HA-Ub with EGFP-Bsn4 was very low and comparable with EGFP control (Fig. 3b). In contrast, we detected a notable HA-Ub association with EGFP-Bsn2 and thereof derived fragments: EGFP-Bsn7 and EGFP-Bsn10 (Fig. 3b). In line with that, endogenous ubiquitin, revealed by FK2 staining, co-localized with cytoplasmic aggregates formed by EGFP-Bsn2 in COS7 cells (Fig. 3c). These results indicate divergent properties of two independent Bsn-PSMB4 interaction interfaces. Bsn2 fragment that interacts with the globular part of PSMB4 and associates with the constituent of the proteasome α-ring, PSMA7, also co-localizes or co-precipitates with mono-and poly-Ub moieties, whereas Bsn4 interacting with the C-terminal part of PSMB4 is not ubiquitinated in HEK293T cells.

To directly assess degradation of Bsn2 and Bsn4 by the UPS, we tested whether pharmacological inhibition of the proteasome using MG132 for 3 or 6 h alters expression levels of Bsn fragments in HEK293T cells. We chose these two specific time points to exclude effects of prolonged proteasome inhibition on protein synthesis and gene expression reported previously [8]. Quantification did not reveal any differences in the accumulation of Bsn2 or Bsn4 upon inhibition of proteasome with MG132 as compared to the overexpressed EGFP suggesting that fusion of EGFP to the PSMB4-binding fragments does not promote their preferential degradation by proteasome (Fig. 3e, h: EGFP: 1.03 ± 0.04, EGFP-Bsn2: 0.99 ± 0.09, EGFP-Bsn4: 0.96 ± 0.04; 6 h: EGFP: 0.95 ± 0.04, EGFP-Bsn2: 1.01 ± 0.11, EGFP-Bsn4: 1.03 ± 0.06). This data indicate that PSMB4-interacting fragments of Bsn are not subjected to proteasomal degradation.

### Bsn inhibits proteasomal activity in HEK293T cells

**Fig. 4:**
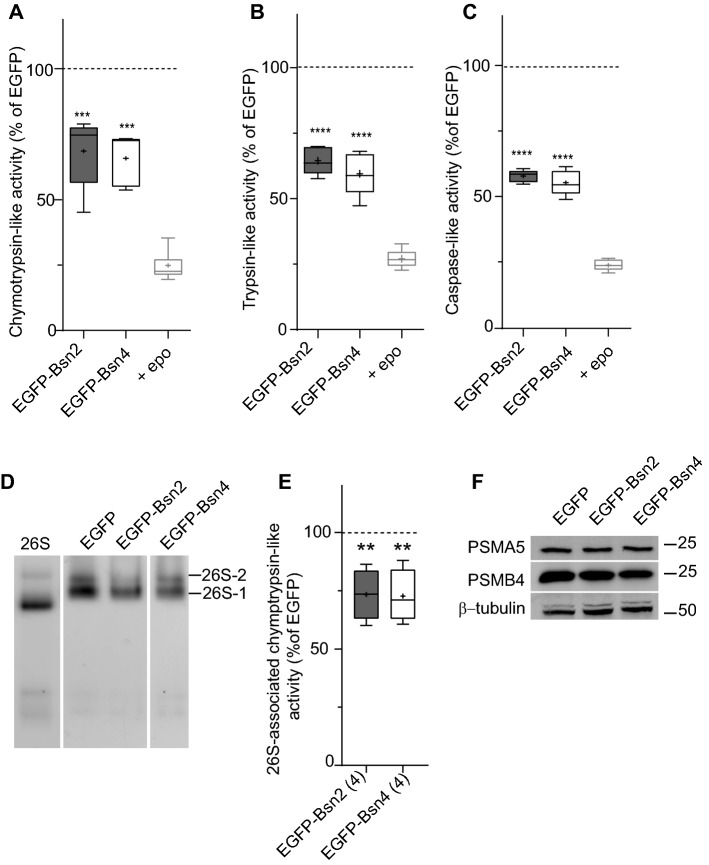
Proteasome activity is altered upon expression of Bsn fragments.Chymotrypsin (**a**), trypsin- (**b**) and caspase-like (**c**) activities were measured in lysates of HEK293T cells expressing EGFP, EGFP-Bsn2 or EGFP-Bsn4 using fluorogenic peptide substrates. Lysates treated with epoxomicin, an irreversible proteasome inhibitor, were always used to validate the assay. **d** 26S proteasomes were resolved using native-PAGE in lysates of HEK293T cells transfected with EGFP, EGFP-Bsn2 or EGFP-Bsn4 and the proteasome-associated activity was assessed by in-gel overlay activity assay (zymography). 26S-2 represents doubly capped proteasome, 26S-1-singly capped proteasome. **e** Densitometric quantification of the experiment from (D) normalized to the 26S proteasome activity in EGFP-transfected cells. **f** Expression levels of proteasome α (PSMA5) and β (PSMB4) subunits were unchanged upon expression of EGFP or Bsn fragments. β-tubulin III served as a loading control. Experiments were conducted three to four times on independent HEK293T cell cultures. Comparisons to (EGFP transfected cells) were obtained by one-way ANOVA test with Dunnett’s posttest

Interaction of Bsn fragments with PSMB4 prompted us to further examine the effect of Bsn2 and Bsn4 expression on the proteasome function. To this end, we overexpressed EGFP, EGFP-Bsn2 or EGFP-Bsn4 in HEK293T cells and measured specific proteolytic activities of the endogenous proteasome in lysates of these cells using fluorogenic peptides designed to monitor chymotrypsin-, trypsin- and caspase-like activities of proteasome. To assess proteasome-independent peptidase activities we performed the same measurement in cell lysates treated with epoxomicin (epo), a potent proteasome inhibitor. The expression of both Bsn2 and Bsn4 significantly reduced all three major hydrolytic activities of the proteasome in the lysates of cells transfected with Bsn2 and Bsn4 as compared to the control cells transfected with EGFP (Fig. 4a–c; in  %, chymotrypsin-like activity: EGFP: 100 ± 1, EGFP-Bsn2: 69 ± 6, EGFP-Bsn4: 67 ± 4 epo: 25 ± 2; trypsin-like activity: EGFP: 100 ± 1, EGFP-Bsn2: 64 ± 2, EGFP-Bsn4: 60 ± 4, epo: 27 ± 1; caspase-like activity: EGFP: 100 ± 1, EGFP-Bsn2: 59 ± 1, EGFP-Bsn4: 56 ± 2, epo: 24 ± 1).

To further confirm that our results reflect modulation of the proteasome-associated endopeptidase activity we subjected lysates of transfected HEK293T cells to native protein acrylamide gel electrophoresis (PAGE), which allows electrophoretic resolving of the native proteasomes. The proteasome-associated chymotrypsin-like activity was visualized by in-gel zymography using Suc-LLVY-AMC substrate and the proteolytic activity was assessed by fluorometric quantification of substrate conversion in the characteristic bands representing native 26S and 20S proteasomes (Fig. 4d). We detected a significantly lower proteasome-associated endopeptidase activity in cells expressing EGFP-Bsn2 and EGFP-Bsn4 compared to control cells transfected with EGFP (Fig. 4e; in  %: EGFP-Bsn2: 74 ± 5, EGFP-Bsn4: 73 ± 6). Western blot analysis of identical lysates did not reveal any changes in the expression of the proteasome subunits PSMA5 or PSMB4 upon expression of Bsn fragments (Fig. 4f; in  %: PSMA5: EGFP-Bsn2: 97 ± 7; EGFP-Bsn4: 90 ± 3; PSMB4: EGFP-Bsn2: 95 ± 19; EGFP-Bsn4: 94 ± 17) suggesting that not regulation of proteasome biosynthesis but rather its assembly or activation are controlled by Bsn.

### Bsn inhibits ubiquitination-dependent and independent proteolysis

**Fig. 5:**
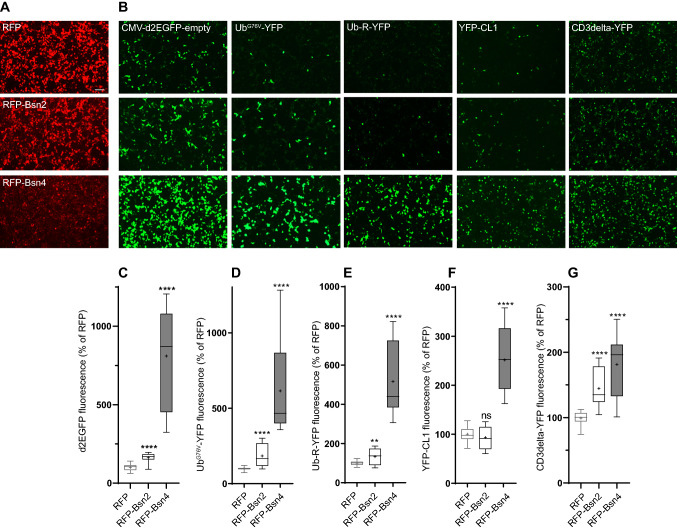
Bsn inhibits protein degradation through ubiquitin-dependent and independent pathways. Representative images of HEK293T cells transfected with RFP (control), RFP-Bsn2, or RFP-Bsn4 (**a**) together with the genetically encoded fluorescent proteasome degradation reporters representing specific substrate classes (**b**). Images were taken 48 h post-transfection. Scale bar 100 μm. **c** Quantification of the EGFP or YFP fluorescence of HEK293T cells expressing RFP, RFP-Bsn2 or RFP-Bsn4 together with different classes of proteasome substrates: ubiquitin-independent d2EGFP (**c**) or soluble Ub-G76V-YFP (d) and Ub-R-YFP (**e**), misfolded cytosolic/nuclear YFP-CL1(F) and ERAD CD3delta-YFP(G) ubiquitin-dependent proteasome substrates. Experiments were performed on three independent HEK293T cell cultures. All values were normalized to the EGFP or YFP expression of the reporter in cells transfected with RFP (control). Statistical significance was assessed by Brown-Forsythe and Welch´s ANOVA test followed by a two step-up method of Benjamini, Krieger and Yekutieli (**c**, **d**, **e**) or by one-way ANOVA followed by Bonferroni´s posttest (F, G) ****p < 0.0001 ** < 0.0021

Fluorogenic peptide substrates bypass ubiquitination reaction and, therefore, do not provide any information about specific routes targeting proteins for degradation by proteasome. To define the effect of Bsn on different pathways, we employed an array of genetically encoded fluorescent proteasome substrates. First, we tested the effect of Bsn fragments on destabilized GFP (d2GFP), where amino acids 422–466 of the degradation domain of mouse ornithine decarboxylase containing a PEST-motif were fused to the C-terminus of EGFP [28]. d2EGFP degradation requires functional 26S proteasome but is ubiquitination independent [21]. The expression of both mRFP-tagged Bsn fragments significantly interfered with the degradation of d2EGFP. Notably, Bsn4 fragment had a much stronger effect than Bsn2 (Fig. 5b, c mRFP-Bsn2: 163 ± 6, mRFP-Bsn4: 810 ± 62 both in  % of RFP). Next, we tested the effect of Bsn expression on the degradation of two soluble cytoplasmic/nuclear ubiquitination-dependent proteasome substrates: Ub^G76V^-YFP, where YFP is fused to an uncleavable Ub moiety, and Ub-R-YFP, which is a N-end rule degradation substrate [7]. Degradation of both Ub^G76V^-YFP and Ub-R-YFP was significantly affected by the expression of Bsn fragments and similarly to what we observed with d2EGFP, also here the effect of Bsn4 was several-fold higher than the effect of Bsn2 (Fig. 5b, d, e; Ub^G76V^-YFP: mRFP-Bsn2: 184 ± 14, mRFP-Bsn4: 615 ± 55; Ub-R-YFP: mRFP-Bsn2: 133 ± 9, mRFP-Bsn4: 517 ± 37; all in  % of RFP). Interestingly, degradation of a misfolded cytosolic/nuclear UPS substrate, YFP-CL1 [14], was affected only upon expression of Bsn4, while Bsn2 had no effect (Fig. 5B,F; mRFP-Bsn2: 93 ± 5, mRFP-Bsn4: 252 ± 13% of RFP). Finally, degradation of CD3delta-YFP, which represents an endoplasmic reticulum-associated degradation (ERAD) substrate [50], was affected to a comparable extent by both Bsn2 and Bsn4 (Fig. 5b, g, mRFP-Bsn2: 146 ± 6, mRFP-Bsn4: 183 ± 9% of RFP).

These experiments revealed that expression of PSMB4-interacting interfaces of Bsn interferes with degradation of different classes of proteasomal substrates. The fact that both ubiquitination-dependent and independent substrates were affected further argues for a direct impact of Bsn on proteasome activity and against secondary effect due to dysregulation of cellular ubiquitination, described earlier in the context of interaction of Bsn with E3-ligase Siah1 [46]. Moreover, a much stronger effect of Bsn4 fragment might be explained by its binding to the C-terminal domain of PSMB4, which is implicated in the 20S proteasome assembly. Thus, the interference of Bsn with proteasome biogenesis might explain the effect of Bsn on the activity of the proteasome.

### Proteasome activity is increased in synaptosomes prepared from brains of Bsn knockout mice

**Fig. 6:**
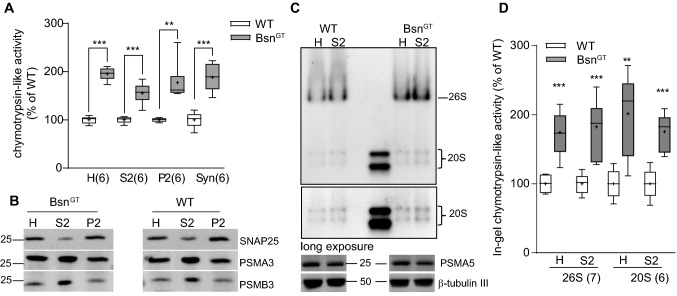
Proteasome activity is significantly upregulated in Bsn knock-out mice. **a** The chymotrypsin-like activity was strongly elevated in the homogenates (H), cytosolic (S1), membrane (P2) and synaptosomal (Syn) brain fractions from Bsn^GT^ mice compared to their wild-type littermates. **b** Expression levels of proteasome subunits PSMA3 and PSMB3 were unchanged between Bsn^GT^ and their WT littermates. An enrichment of t-SNARE SNAP25 in P2 validates successful fractionation. **c** Native proteasomes were resolved in samples of homogenates and cytosolic fractions from brains of Bsn^GT^ mice and their littermates on native-PAGE. Zymography using Suc-LLVY-AMC substrate was used for visualization of the proteasome-associated activity. Expression of PSMA5 was unchanged between genotypes, β-tubulin III served as a loading control. **d** Quantification of 26S and 20S-associated chymotrypsin-like endopetidase activity in **c**. n number corresponds to the number of independently processed fractions from three individual experiments with independent animal material. *p*-values versus control (WT) were obtained by unpaired *t* test

Heterologous expression of Bsn2 and Bsn4 impeded proteasomal activity and degradation of proteasomal substrates in HEK293T cells. To address the role of Bsn in the UPS regulation in neuronal context, we performed subcellular fractionation of brain tissue from mice lacking functional expression of Bsn (Bsn^GT^) [17] and their wild-type (WT) littermates and measured chymotrypsin-like endopeptidase activity using fluorimetry of Suc-LLVY-AMC in these samples. We detected a significant increase in the chymotrypsin-like activity in the crude brain homogenate, cytosol (S2) as well as in the crude membrane (P2) and synaptosomal (Syn) fraction samples from Bsn^GT^ as compared to WT, indicating an upregulation of proteasome activity in the synaptic compartment in the absence of Bsn (Fig. 6a; homogenate: 195 ± 6, S2: 156 ± 9, P2: 177 ± 17, Syn: 188 ± 12% of WT). Immunoblotting with antibodies against the core proteasomal subunits PSMA3 and PSMB3 confirmed comparable expression of proteasome subunits in WT and Bsn^GT^ in all tested fractions (Fig. 6b). Next, we subjected samples from the fractionation to native- PAGE followed by in-gel zymography for chymotrypsin-like endopeptidase activity. We found that peptidase activity associated with both 20S and 26S proteasome was considerably higher in the total brain lysates and in the cytosolic fractions from Bsn^GT^ mice (Fig. 6c, d; 26S: homogenate: 175 ± 12, S2: 183 ± 15; 20S: homogenate: 202 ± 24%, S2: 175 ± 11% of WT). Also in these samples, quantitative immunoblotting did not reveal significant changes in the expression levels of the proteasomal subunit PSMA5 in Bsn^GT^, suggesting that modulation of the proteasome assembly or activity, but not its expression, underlies increased proteasome activity in Bsn knock-out mice (Fig. 6c; PSMA5: H: 84 ± 12, S2: 88 ± 17% of WT).

### Expression of PSMB4-interacting fragments of Bsn normalizes increased proteasome activity in Bsn^GT^

**Fig. 7:**
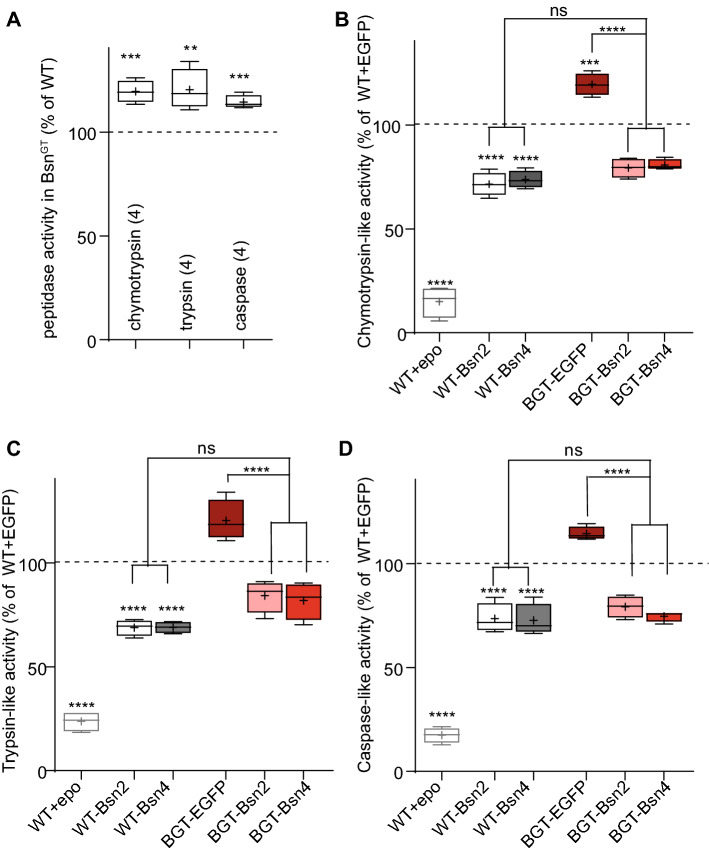
Overexpresion of the PSBM4-interacting interfaces of Bsn in Bsn^GT^ cultures rescues the proteasome activity levels. **a** Chymotrypsin-, trypsin- and caspase-like activities are elevated in Bsn^GT^ cortical neurons as compared to the neurons from their WT littermates. Chymotrypsin- (**b**), trypsin- (**c**) and caspase-like (**d**) activities were significantly reduced upon expression of PSMB4-interacting fragments EGFP-Bsn2 or EGFP-Bsn4 in WT neurons. Importantly, there was no difference in proteasome activities between WT and Bsn^GT^ neurons expressing EGFP-Bsn2 or EGFP-Bsn4. Treatment with epoxomicin was always performed to validate the activity assay. In A, n number (in parentheses) denotes the number of experiments performed on independently prepared cultures. *p*-values versus control (WT-EGFP) were obtained by unpaired *t* test. Data are represented as boxes indicating the interquartile distance with median, whiskers minimum and maximum values, and mean showed as +. In **b**, **c** and **d** *p* values versus control (WT-EGFP) were obtained by one-way ANOVA with Tukey’s posttest

Bsn-deficient mice display systemic phenotypes including epilepsy and might be also prone to neurodegeneration [2, 13, 41, 49]. To determine whether increased proteasome activity is a direct result of bassoon deficiency or a secondary effect of systemic phenotypes described in the animals deficient for Bsn expression, we tested chymotrypsin-, trypsin and caspase-like peptidase activities using fluorogenic specific peptidase substrates in cultured cortical neurons derived from newborn Bsn^GT^ animals and their WT littermates. All tested peptidase activities associated with proteasome were significantly increased in cell lysates from cultured neurons from Bsn^GT^ animals compared with their WT littermates (Fig. 7A and B-D carmine box; chymotrypsin-like activity: Bsn^GT^-EGFP: 120 ± 3, trypsin-like activity: Bsn^GT^-EGFP: 121 ± 5 and caspase-like activity: Bsn^GT^-EGFP: 115 ± 2% of WT-EGFP). Similarly to HEK293T cells (Fig. 4a–c), expression of fragments Bsn2 and Bsn4 significantly decreased all tested proteasome-associated peptidase activities in WT neurons indicating a physiological role of the PSMB4-interacting interfaces of Bsn in the regulation of the endogenous neuronal proteasome (Fig. 7b–d, white and gray boxes, chymotrypsin-like activity: WT-Bsn2: 72 ± 3, WT-Bsn4: 74 ± 2 of WT-EGFP, WT-EGFP epo: 15 ± 4; trypsin-like activity: WT-Bsn2: 69 ± 2, WT-Bsn4: 70 ± 1, WT-EGFP epo: 24 ± 2; caspase-like activity: WT-Bsn2: 74 ± 4, WT-Bsn4: 73 ± 4% of WT-EGFP, WT-EGFP epo: 17 ± 2, all in  % of WT-EGFP). Strikingly, expression of Bsn2 and Bsn4 effectively reduced and fully normalized the elevated peptidase activities in Bsn^GT^ neurons (Fig. 7b–d, pink and red boxes chymotrypsin-like activity: Bsn^GT^-Bsn2: 79 ± 2, Bsn^GT^-Bsn4: 81 ± 1; trypsin-like activity: Bsn^GT^-Bsn2: 84 ± 4, Bsn^GT^-Bsn4: 82 ± 5; caspase-like activity: Bsn^GT^-Bsn2: 79 ± 3, Bsn^GT^-Bsn4: 75 ± 1, all in  % of WT-EGFP). This data confirms a key role of Bsn-PSMB4 multivalent interaction in the regulation of proteasome activity in neurons. Moreover, it strongly indicates that increased proteasome activity detected upon deletion of Bsn can be attributed to this molecular interaction.

### Bassoon controls the abundance of presynaptic substrates of proteasome via its PSMB-4 binding interface

**Fig. 8:**
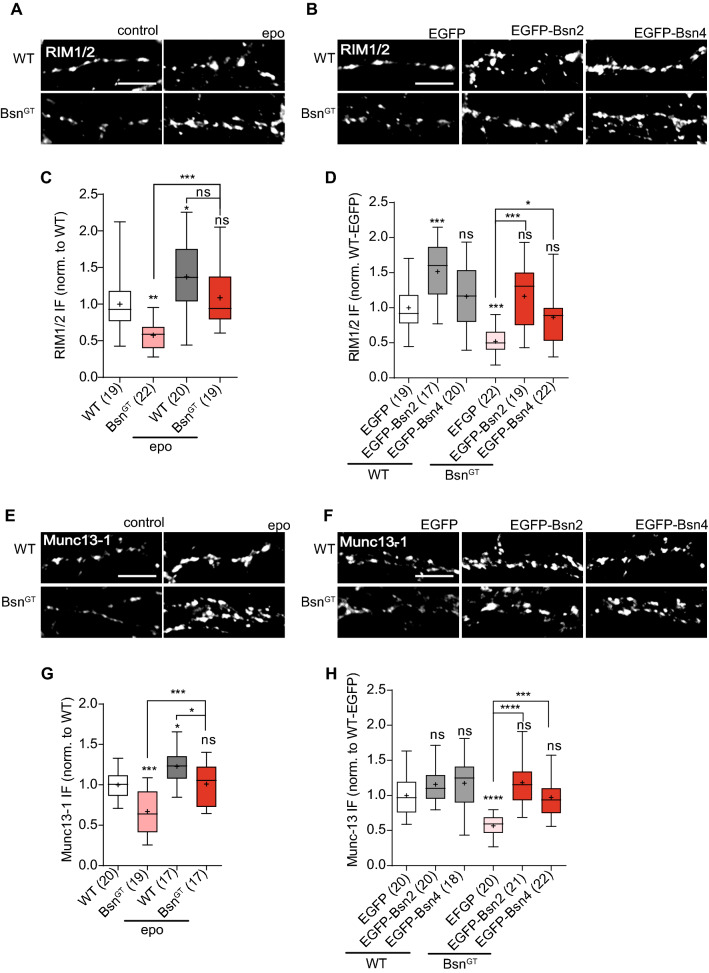
Expression levels of presynaptic proteasome substrates are decreased in Bsn^GT^ mice and normalised upon expression of PSMB4-interacting interfaces.Representative images of 15 DIV hippocampal neurons from WT and Bsn^GT^ mice in basal conditions and treated with epoxomicin (**a**, **e**) or transduced with EGFP, Bsn2-EGFP, Bsn4-EGFP(**b**, **f**) and stained for RIM1/2 (**a**, **b**) or Munc13-1 (E,F). Scale bar 5 μm. Epoxomycin increased synaptic RIM1/2 (**c**) and Munc13-1 (**g**) in both WT and Bsn^GT^ neurons. Expression of Bsn2-GFP and Bsn4-GFP largely normalised synaptic levels of RIM1/2 (**d**) and Munc13-1 (**h**) in Bsn^GT^ neurons. Analyses were performed on primary hippocampal cultures prepared independently from 4 animals per genotype. In graphs, n numbers (in parentheses) show the number of analysed cells. *p* values were obtained by one-way ANOVA with Tukey’s posttest and are depicted above each box for comparison to the control group and above the brackets for comparison with untreated Bsn^GT^ neurons

Presynaptic scaffolding proteins Rab3-interacting molecules (RIMs) and Munc13s (mammalian homolog of unc-13), are documented targets of UPS-dependent degradation at presynapse [43, 51]. To directly assess the possibility of regulation of proteasome-dependent protein degradation within the presynaptic compartment via Bassoon-PSMB4 interaction, we investigated the effect of Bassoon depletion on the synaptic abundance of presynaptic proteins RIM1/2 and Munc13-1. To this end, we performed quantitative immunostaining in hippocampal neurons of WT and Bsn^GT^ mice in the basal state and upon treatment with epoxomicin, a potent proteasome blocker (0.1 µM, 16 h) (Fig. 8a, c, e, g). Additionally, PSMB4-interacting fragments Bsn2 and Bsn4 were overexpressed in WT and Bsn^GT^ neurons (Fig. 8b, d, f, h). Synaptic levels of RIM1/2 and Munc13 were significantly lower in the Bsn^GT^ compared to the WT and treatment with epoxomicin (epo) resulted in a rise in synaptic expression levels, confirming regulation of synaptic RIMs and Munc13-1 by proteasome in both genotypes (Fig. 8c and G; RIM 1/2: Bsn^GT^: 0,57 ± 0.04, Bsn^GT^ epo: 1.09 ± 0.09, WT-epo: 1.37 ± 0.10; Munc13-1: Bsn^GT^: 0.67 ± 0.06, Bsn^GT^ epo: 1.01 ± 0.06, WT-epo: 1.23 ± 0.10, mean ± SEM, all normalized to WT). Notably, the blockage of proteasome substantially equalized the levels of RIM1/2 and Munc13-1 in WT and Bsn^GT^ neurons, indicating a causal role of elevated proteasomal activity in absence of Bsn for the depletion of these synaptic components. Expression of Bsn2 and Bsn4 fragments increased RIMs and Munc13-1 in both WT and Bsn^GT^ neurons, further supporting the role of Bsn-PSMB4 interaction in the regulation of RIM1/2 and Munc13-1 turnover at presynapse (Fig. 8d and h; RIM1/2: WT-Bsn2: 1.51 ± 0.11, WT-Bsn4: 1.16 ± 0.10, Bsn^GT^ -EGFP: 0.52 ± 0.04, Bsn^GT^-Bsn2: 1.16 ± 0.09, Bsn^GT^-Bsn4: 0.87 ± 0.08; Munc13-1: WT-Bsn2: 1.16 ± 0.06, WT-Bsn4: 1.17 ± 0.09, Bsn^GT^ -EGFP: 0.57 ± 0.04, Bsn^GT^-Bsn2: 1.18 ± 0.07, Bsn^GT^-Bsn4: 0.97 ± 0.06, all normalized to WT-EGFP). This further supports a notion that Bsn is able to control the rate of proteasomal degradation of presynaptic components via its direct interaction with PSMB4.

## Discussion

### Bassoon binds PSMB4 via multiple functionally distinct interfaces

Here, we describe a novel interaction between Bsn and PSMB4, the β7 structural subunit of the 20S core proteasome. We identified three independent PSMB4-binding interfaces on Bsn that differ not only in their binding site on PSMB4 but also in their effect on proteasome activity and on proteolysis of different classes of proteasome substrates. Bsn2 and its subfragments Bsn7 and Bsn10, which are located upstream of the second coiled-coil (CC2) domain, interacted with the globular part of PSMB4. Bsn4 fragment, localized downstream of CC2 domain, associated specifically with C-terminal region of PSMB4 (Fig. 2). The active proteasome is made of two half proteasomes each composed of an α- and a β-ring. PSMB4 incorporates as the last subunit of the β-ring and its arrival is tightly coupled with dimerization of two inactive half-proteasomes leading to the assembly of the proteolytically competent 20S particle. Intercalation of the extended C-terminal region of PSMB4 into the opposing half-particle is the structural determinant of this rate-limiting step in the proteasome biogenesis [4, 38]. We observed an interaction of Bsn4 with this 14 amino acid-long region of PSMB4 and considering the compact structure of the proteasome barrel, this interaction is likely to interfere with the normal dimerization of the half-proteasomes and ultimately with 20S proteasome assembly. In line with these structural constraints, expression of Bsn2 and Bsn4 interfered with the degradation of proteasome substrates in cells. While the degradation of all soluble cytoplasmic substrates was strongly affected by the expression of Bsn4, the expression of misfolded cytoplasmic CL1 degron-containing proteins and ERAD-substrate was less affected. Interestingly, both CL1 degron and ERAD substrates are targeted by an ER-associated degradation machinery [33], which as our data indicate, appears less sensitive to regulations by Bsn compared to degradation of soluble proteasome substrates. Similar to Bsn4, expression of Bsn2, significantly impeded degradation of soluble proteasome substrates and the ERAD-substrate, however, too much lesser extent than Bsn4. This reveals a key functional importance of Bsn4 interaction with the C-terminus of PSMB4. Importantly, expression of PSMB4-interacting Bsn fragments affected the degradation of both ubiquitination-dependent and independent soluble substrates indicating that Bsn controls proteasome activity by an ubiquitination-independent mechanism. Moreover, Bsn2 and thereof derived fragments Bsn7 and Bsn10, co-precipitated or co-localized with ubiquitinated moieties and other structural subunits of the proteasome suggesting their ability to associate with assembled proteasome species. However, this needs to be addressed by further experiments.

Our interactions assays revealed that Bsn possess three structurally and functionally independent moieties that interact and functionally interfere with the core proteasome. Existence of multivalent interactions between binding partners is a common feature allowing dynamic regulation of multiprotein complexes and has been described for multiple interactors of Bsn [11, 15, 23]. It is likely that the three binding interfaces of Bsn act in concert to regulate neuronal proteasome. Since Bsn localizes exclusively to the presynaptic active zones, it is conceivable that Bsn assists in dynamic assembly or recruitment of proteasomes species specific for this synaptic subcompartment. In line with this assumption, we observed presynapse-specific effects upon Bsn depletion or upon Bsn2 and Bsn4 overexpression in cultured primary neurons. Specifically, we measured an increased proteasome activity in synaptosomes prepared from brains of Bsn^GT^ mice and in cultured neurons from these animals. Moreover, RIM1/2 and Munc13-1, two exclusively presynaptic UPS substrates were less abundant in synapses of Bsn^GT^ mice cultured in vitro. Importantly, inhibition of proteasome or expression of the PSMB4-bining interfaces of Bsn in Bsn^GT^ neurons normalized both increased proteasome activity and decreased synaptic levels of RIM1/2 and Munc13-1 strongly supporting the role of Bsn-PSMB4 interaction in the modulation of proteasome activity at presynapse and consequently in the regulation of turnover of presynaptic proteins by the proteasome.

### Bassoon regulates multiple neuronal degradation pathways

Recent studies have linked Bsn to multiple pathways involved in the regulation of neuronal proteostasis. Increased neuronal autophagy detected in neurons from Bsn KO mice was attributed to the interaction of Bsn with Atg5, the E3-like ligase that conjugates LC3 to phagophore membranes during autophagosome formation [36]. Earlier, Bsn has been also reported to bind and inhibit the activity of Siah1, which is a part of the SCF E3 Ub-ligase complex involved in the degradation of SV proteins through ESCRT/endo-lysosomal system [46]. Importantly, Bsn fragments that interact with PSMB4 and inhibit proteasomal activity do not overlap with binding sites for Atg5 or Siah1. This indicates that the functional regulation of proteasome activity described here is not a secondary effect of altered cellular ubiquitination or autophagy pathway.

Thus, Bsn controls neuronal proteostasis by multiple pathways via multiple autonomous interactions. Despite their independent molecular basis, Bsn-based regulation of proteasomal, endo/lysosomal and autophagosomal systems likely intersect in neurons. Crosstalk between these degradation pathways is starting to emerge but the molecular mechanisms of these cross- regulations remain elusive [47]. Proteins like Bsn linking multiple proteostasis pathways might represent novel regulatory nodes. In this context, Cereblon (Crbn), a brain protein implicated in mental retardation and the target of thalidomide teratogenity, shows some interesting parallels [3, 18]. Similar to Bsn, Crbn binds PSMB4 and inhibits proteasome activity as well as degradation of Ub-conjugated proteins [26]. Crbn also associates with E3 Ub-ligase complex-CRL4A modulating its activity and substrate specificity [22], and regulates autophagy [25, 35]. Thus, convergent evolution of neuronal proteins that regulate proteasome activity, protein ubiquitination and autophagy might reflect the specific need for coupling of these processes in neuronal cells, which is an important issue for future studies.

### Bsn and protein aggregation -induced neuropathy

Recently, mutations in BSN have been linked to human diseases associated with pathological protein aggregation. Bsn mutation found in the patients with familiar PSP-like syndrome significantly decreased the solubility and degradation of Bassoon in heterologous expression system and importantly, led to increased tau aggregation [49]. Moreover, highly upregulated Bsn expression and its accumulation in large aggregates in the somata of motoneurons, which is a characteristic hallmark of the inflammation-induced degeneration, were found in spinal cord samples from patients with multiple sclerosis (MS) and from the respective disease mouse model, the experimental autoimmune encephalomyelitis (EAE). Strikingly, Bsn knock out animals were protected from the inflammation-induced formation of these toxic somatic aggregates, displayed lower motoneuron degeneration and milder clinical disability in EAE. The expressional profiling indicated aberrant protein homoeostasis in MS patients and EAE mice and pharmacological increase of UPS activity in EAE mice had a similar effect as Bsn deletion [41]. In this work, we demonstrate that the multivalent interaction of Bsn with PSMB4, the structural subunit of 20S proteasome, directly inhibits overall proteasome activity, which offers a molecular explanation for the negative impact of Bsn expression on neurodegeneration in EAE. It is feasible that somatic Bsn aggregates formed in motoneurons in MS and EAE excessively inhibit the somatic proteasome activity and thus, drive failure of protein turnover leading to aggregation-induced neurodegeneration. Future studies should elucidate the conditions under which Bassoon binds PSMB4 at the synapse and how this interaction affects synaptic function.

## Materials and methods

### Antibodies and reagents

For immunocytochemical staining (ICC) and Western blotting (WB) the following primary antibodies were used: mouse antibodies against flag (ICC and WB 1:1000, Sigma-Aldrich, F1804), SNAP25 (WB 1:1000, Synaptic Systems, 111 011), β-actin (WB 1:1000, Sigma), PSMA7 (ICC and WB 1:1000, MCP34; PW8120), PSMA5 (WB 1:1000, MCP196), PSMA6 (WB 1:1000, MCP20; PW8100), PSMA3 (WB 1:1000, MCP72; PW8110), PSMB7 (WB 1:1000, MCP168; PW8145), PSMB3 (WB 1:1000, MCP102), PSMB4 (WB 1:1000, MCP205; PW8135),

PSMC5 (WB 1:1000, p45-110) and FK2 (ICC and WB 1:1000; PW8810). All antibodies against proteasome subunits were purchased from Enzo Life Sciences. Rabbit antibodies against RIM 1,2 (ICC 1:500, Synaptic System, # 140203), Munc13-1(ICC 1:1000, Synaptic System, # 126103), β- tubulin III (WB 1:1000, Sigma-Aldrich), PSMG1/PAC1 (WB 1:1000, Cell Signaling, #13378), PSMC4 (WB 1:1000, Bethyl Labolatories Inc), GFP (WB 1:1000, Abcam, ab 6556), RFP (ICC and WB 1:1000, Rockland Immunochemicals Inc.). Secondary antibodies from goat or donkey coupled with Cy3 (ICC 1:1000) or peroxidase- (1:10000) were obtained from Jackson ImmunoResearch Laboratories. MG132 and epoxomicin were purchased from Enzo Life Sciences.

### Animals

Cells and tissues used in the study were obtained from Wistar rats and bassoon gene trap (Bsn^GT^) [17] mouse strains backcrossed to C57BL/6 N. Bsn^GT^ mice were obtained from Omnibank ES cell line OST486029 by Lexicon Pharmaceuticals, Inc. (The Woodlands, TX). All experiments were performed in accordance with the European Committees Council Directive (86/609/EEC) and approved by the local animal care committee (Landesverwaltungsamt Sachsen-Anhalt, AZ: 42502-2-1303 LIN).

### DNA constructs

N-terminally truncated PSMB4 covering nucleotides (nt) 118-820 and amino acids (aa) 32- 264, 26 kDa, ΔNPSMB4: nt 161–820, aa 45–264, 24 kDa as well as ΔNΔC-PSMB4: nt 160–764, aa 45–246, 22 kDa of rat Psmb4 (NM_031629.2, NP_113817.2) were produced by PCR using the pACT2 rat brain Matchmaker cDNA library (Clontech Laboratories, Inc.) as a template with extended primers, adding EcoRI and XhoI restriction sites at the 5′ and 3′ ends of the fragments, respectively. The introduced restriction sites were used for in-frame cloning of the fragments into the pCMV-Tag2B and pCMV-Tag3B vectors (Agilent Technologies). mRFP-PSMB4 was created by insertion of N-terminally truncated PSMB4 (aa 32–264) into mRFP-C2 vector that was generated as previously described [11] RFP-CtermPSMB4 (nt 767–820, aa 247–264, 2 kDa) construct was created by insertion of annealed synthetic oligonucleotides into pmRFP-C2 vector. Bsn fragments Bsn4 (aa 2715–3013, 33 kDa) and Bsn1 (aa 1692–3263, 169 kDa) [9] as well as Bsn2 (aa 1653–2082, 47 kDa) [44] were described previously. pEGFP-Bsn2 and pEGFP-Bsn4 were subcloned into RFP-C2 vector as well as FUGW lentiviral transfer vector [29]. Bsn5 (aa 2715–2820 of NP_062019.2, 11 kDa) was created from pEGFP-Bsn4 as a template using PCR with extended primers. EcoRI and BamHI sites were introduced at 5′ and 3′ ends, respectively, and used for in-frame cloning of the fragment into the pBS-SK (+) and pEGFP-C2 vector. Bsn3 and Bsn6 were generated using PCR on rat cDNA of Bsn as a template. EcoRI and XhoI restriction sites were added at the 5′ and 3′ ends of the fragments, respectively. Bsn constructs Bsn7 (aa 1653–1878, 24 kDa), Bsn8 (aa 1964–2087, 14 kDa), Bsn9 (aa 1653–1963, 33 kDa) and Bsn10 (aa 2013–2087, 8 kDa) were generated using PCR on Bsn2 as a template, with extended primers to add EcoRI and XhoI (Bsn9, Bsn7, and Bsn8) or EcoRV and XhoI (Bsn10) restriction sites at the 5′ and 3′ ends of the fragments, which were used for in-frame cloning of fragments into pGADT7, pCMV-3B or pBS-SK(+) vectors. All the fragments were also inserted into pEGFP-C2 vector. All constructs were verified by sequencing. HA-Ubiquitin (#18712) [24], Ub-G76V-YFP (#11949), Ub-R-YFP (#11948),), CD3delta YFP (#11951) [32] and CMV-d2EGFP-empty (#26164) [10], were purchased from Addgene.

### Primary neuronal cultures

Primary cultures of hippocampal and cortical neurons from P0 to P1 Bsn^GT^ mice and their wild-type siblings were prepared as described previously [1]. Briefly, after trypsin treatment of the hippocampus and mechanical trituration, cells were plated in densities of 3.5*10^4^ cells per coverslip (18 mm diameter). 1 h after plating, coverslips were transferred into dishes containing 60–70% confluent monolayer of astrocytes and Neurobasal A medium supplemented with B27, 1 mM sodium pyruvate, 4 mM Glutamax and antibiotics (100U/ml penicillin, 100ug/ml streptomycin). At 1 and 3 days in vitro (DIV) AraC (Sigma Aldrich) was added to the cells (0,6 µM each time) to reach a final concentration of 1.2 µM. For cortical culture preparation, the brains were dissected, and meninges removed. Tissue was treated with 0.25% trypsin for 20 min and triturated in the presence of 0.1% DNAse. 2*10^6^ cells were plated in DMEM with 10% FCS (fetal calf serum), 1 mM glutamine and antibiotics (100U/ml penicillin, 100 µg/ml streptomycin, Life Technologies) into poly-d-lysine coated 75 cm^2^ flasks. After 7–8 h the medium was changed to Neurobasal A supplemented with B27, 1 mM sodium pyruvate, 4 mM Glutamax and antibiotics (100 U/ml penicillin, 10 μg/ml streptomycin). At 4 DIV 0.6 μM AraC was added.

### Lentiviral particles production and infection of neuronal cells

Lentiviral particles were generated in HEK293T cells (ATTC, Manassas, VA, USA) using FUGW-based transfer, psPAX2 packaging and pVSVG pseudotyping vectors [29]. HEK293T cells were grown in media containing 10% FCS to 60% confluence in the 75 cm^2^ flasks. Cells were transfected with 20 µg of total DNA per flask using calcium phosphate method [11]. Molar ratio of FUGW: psPAX2: pVSVG was 2:1:1. 6–8 h after transfection, medium was changed to 10 ml production medium containing Neurobasal A supplemented with antibiotics, 1 mM sodium pyruvate (Life Technologies), B27 and 1 mM Glutamax (Life Technologies). 48 h after transfection, virus-containing media was collected and cleared from large cellular debris by centrifugation for 20 min at 2000*g*. Virus-containing supernatant was aliquoted and stored at − 80 °C. For infection of hippocampal and cortical cultures, viral particles were applied overnight at 4 DIV. Neurons were stained or collected for proteasome activity assays at 14–18 DIV.

### Immunoprecipitation and Western blotting

HEK293T cells were transfected using the standard calcium phosphate method [11]. One day after transfection, cells were lysed in 50 mM Tris–HCl, pH 8.0, 150 mM NaCl, 1% Triton X-100, complemented with complete protease inhibitor (Roche) for 10 min on ice and cleared by centrifugation for 10 min at 15,000 g. Co-immunoprecipitation was performed using MicroMACS anti-GFP Microbeads and Micro Columns (Miltenyi Biotec) according to the manufacturer’s instructions, except for the washing steps where lysis buffer was used. Bound proteins were eluted in the SDS-loading buffer, heated for 5 min at 95 °C and analyzed by immunoblotting. Briefly, the samples were separated on 5%-20% Tris- glycine gradient polyacrylamide gels and blotted onto PVDF membrane (Millipore) by wet electroblotting system (Hoefer). The membranes were incubated with indicated antibodies diluted in PBS containing either 5% BSA or 5% non-fat dry milk and supplemented with 0.1% Tween-20. Immunodetection was performed using Pierce ECL WB Substrate (Thermo Scientific) and ChemoCam Imager (Intas). Size markers in Western blot images are indicated in kDa.

### Co-recruitment assay in COS7 cells

COS-7 cells grown on the glass coverslips were transfected using Polyfect reagent (QIAGEN) according to the manufacturer’s protocol. After 24 h, cells were fixed, blocked and stained as described elsewhere [11]. Images were acquired with Zeiss Axio Imager A2 microscope with Cool Snap EZ camera and VisiView Software (Visitron Systems).

### Mouse brain fractionation

For the preparation of subcellular fractions, 6–7 weeks mice were sacrificed by cervical fracture. Cortex and hippocampi were dissected and homogenized in the buffer containing 0.32 M sucrose, 5 mM Tris–HCl, pH 7.4, 5 mM MgCl_2_, 1 mM DTT and 2 mM ATP, with a Potter glass-Teflon homogenizer using 12 strokes at 900 rpm. This and all the following procedures were carried out at 4 °C. The homogenate (H) was centrifuged at 1000*g* for 10 min to sediment nuclear fraction and cell debris (P1). The supernatant (S1) was collected and centrifuged at 10,000*g* for 15 min, yielding pellet (P2, membrane-enriched fraction) and supernatant (S2, cytosol). For isolation of synaptosomes (Syn), the P2 fraction was resuspended in a buffer containing 0.32 M sucrose, 5 mM Tris–HCl pH 8.0 and 1 mM ATP, laid atop a discontinuous sucrose gradient (0.8/1.0/1.2 M; 3 ml per step) and centrifuged for 2 h at 85,000*g*. The Syn fraction was collected from the 1.0/1.2 M sucrose interface.

### Chymotrypsin, Caspase and Trypsin-like peptidase activity assay on lysates from HEK293T cells, cortical neurons, or brain samples

One day after transfection or 14–16 days after infection, HEK293T cells or mice cortical neurons, respectively, were lysed in 50 mM Tris–HCl, pH 7.5, 250 mM sucrose, 5 mM MgCl_2_, 1 mM DTT, 2 mM ATP, 0.5 mM EDTA, 0.025% digitonin for 10 min at 4 °C. The lysate was cleared by centrifugation for 15 min at 20,000 g. Protein concentration was measured by Coomassie Plus Bradford Assay according to manufacturer instructions (Thermo Scientific). Protein concentration after subcellular brain fractionation was determined using the BCA kit (Pierce BSA). For the assessment of the proteasome activity from HEK239T cells or cortical mice cultures, equal amounts of total protein (5 μg/100 µl well) were incubated with the assay buffer (50 mM Tris–HCl, pH 7.5, 5 mM MgCl_2_, 40 mM KCl, 1 mM DTT, 2 mM ATP) containing 100 μM N- Succinyl-Leu-Leu-Val-Tyr-7-amino-4-methylcoumarin (Suc-LLVY-AMC) for chymotrypsin-like activity, 100 μM Z-LLE-7-Amino-4-methylcoumarin (Z-Leu-Leu-Glu-AMC) for Caspase-like activity and 100 μM Boc-LRR-7-Amino-4-methylcoumarin (Boc-Leu-Arg–Arg-AMC) for Trypsin-like activity (all fluorogenic peptides were purchased from Enzo Life Science). For determination of the chymotrypsin-like proteasome activity in different brain fractions, equal amounts of protein extracts (5 μg/100 µl well) were incubated with the assay buffer (0.5 mM EDTA, 50 mM Tris–HCl, pH 8) containing 40 μM Suc-LLVY-AMC [42]. 30 min at 37 °C and the proteasome activity was recorded as a fluorescent signal of AMC release. Fluorescence (380 nm/440 nm excitation/emission) was detected using the Fluostar Omega microplate reader with appropriate fluorescence filters (BMG Labtech). The assays were performed in quintupled for HEK cells and in quadruplicate for cortical neurons. After background subtraction from each data point, the values were normalized to the proteasome activity of control-GFP transfected HEK cells or WT FUGW-GFP infected neurons and expressed in  %. Treatment with 20 μM Epoxomicin for 30 min at 37 °C was used to verify the validity of the assay.

### Native gel electrophoresis and in-gel activity assay (zymography)

To resolve proteasome complexes either HEK293T cell lysate (25 mM Tris–HCl, pH 7.5, 5 mM MgCl2, 1 mM DTT, 2 mM ATP, 0.025% digitonin) or brain fraction lysate were subjected to the native gel electrophoresis. 50 μg of the brain fractions lysate or 20 µg of HEK293T cell lysate was separated on 3%-8% NuPAGE Tris–Acetate Mini Gels (Life Technologies). The gels were run at 4 °C, first at 150 V for 1.30 h, thereafter, the voltage was increased to 200 V for the next 2.30 h. The in-gel activity of the 26S proteasome was revealed by the incubation of the gel in the buffer (20 mM Tris–HCl, pH 7.5, 5 mM MgCl_2_, 2 mM ATP) containing 100 μM Suc-LLVY-AMC for 20 min at 37 °C. Proteasome activity was detected upon illumination with UV light (excitation 366 nm, emission 440/40 nm) using ChemoCam Imager (Intas). Fluorescence intensity was quantitated using ImageJ software.

### Measurement of the UPS activity in HEK293T cells using fluorescent proteasome substrates

HEK293T cells were co-transfected at 70% confluency with an equimolar amount of each fluorescent UPS substrates together with RFP or RFP-tagged Bsn constructs using the jetPEI (Polyplus) transfection reagent according to the manufacturer´s instructions.48 h post-transfection, cells were resuspended, counted and plated at the density of 70,000 cells/well in 96-well poly-l-lysine-coated clear-bottom black well plates (Costar). YFP or GFP fluorescence was measured in a Clariostar microplate reader (BMG Labtech) (YFP: excitation 497-15; emission 540-20; GFP: excitation 470-15; emission 515-20) one day later. The background fluorescence (i.e. mean fluorescence of non-transfected cells) was subtracted from each raw data. Eight wells were quantified for each condition and all data were normalized to the control (RFP) and expressed in percentage.

### Quantitative immunostaining, image acquisition and analysis

Cultured hippocampal neurons from newborn WT and Bsn^GT^ mice were fixed in 4% paraformaldehyde (PFA), 4% sucrose in PBS for 5 min and washed twice with PBS. Then, cells were permeabilized for 30 min with blocking solution (10% FCS, 0.1% glycine and 0.3% Triton X-100 in PBS). Primary antibodies were applied overnight at 4 °C. Afterwards, cells were washed four times with PBS (10 min each) and incubated with secondary antibodies for 1 h at room temperature. Both primary and secondary antibodies were diluted in PBS containing 3% FCS. Finally, coverslips were dipped in water and mounted on glass slices with FluorSave™ (Calbiochem). Images of stainings were acquired on a Zeiss Axio Imager A2 microscope with Cool Snap EZ camera (Visitron Systems) controlled by VisiView (Visitron Systems GmbH) software. For quantifications, settings of camera were applied identically to all coverslips quantified in one experiment. For each IF quantification in each experiment, images from at least two different coverslips (5–7 cells each) were acquired and quantified to avoid effects given by experimental variance. Unspecific background was removed using threshold subtraction in ImageJ software (NIH, http://rsb.info.nih.gov/ij/). In all experiments, synaptic puncta were defined semiautomatically by setting rectangular regions of interest (ROI) with dimensions of about 0.8 X 0.8 um around local intensity maxima in the channel with staining for RIMs, and Munc13-1 using OpenView software (written and kindly provided by N.E. Ziv [45]. Mean IF intensities were measured in synaptic ROIs in all corresponding channels using the same software and normalized to the mean IF intensities of the control group for each of the experiments.

### Statistical analysis

All the results of quantitative analyses are expressed as means ± standard errors of the mean (s.e.m) and in graphs, represented as boxes indicating the interquartile distance with median, whiskers minimum and maximum values with mean showed as +. Statistical analysis was done with Prism 8 software (GraphPad Software, Inc.), Test were used as indicated specifically for each experiment. The normal distribution of the data was verified before choosing the appropriate test. In all graphs, numbers within bars depict the number of independent values used for statistics. Statistical significance is marked as non-significant (ns) or with stars as follows: *p < 0.0332, ** or ^##^*p* < 0.0021, *** or ^###^*p* < 0.0002 ****p < 0.0001, in the graphs.

### Electronic supplementary material

Below is the link to the electronic supplementary material.

Supplementary material 1 (PDF 1492 kb)
