# Supplementary figures and images for: Bassoon inhibits proteasome activity via interaction with PSMB4
Source: Cell Mol Life Sci. 2020 Jul 10;78(4):1545–63. doi: 10.1007/s00018-020-03590-z (PMC7904567; doi:10.1007/s00018-020-03590-z)

**A**

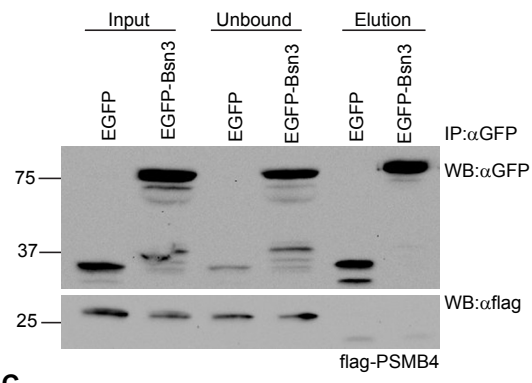

**B**

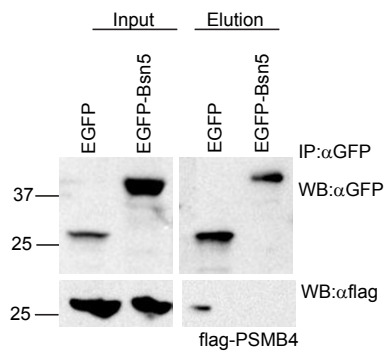

**C**

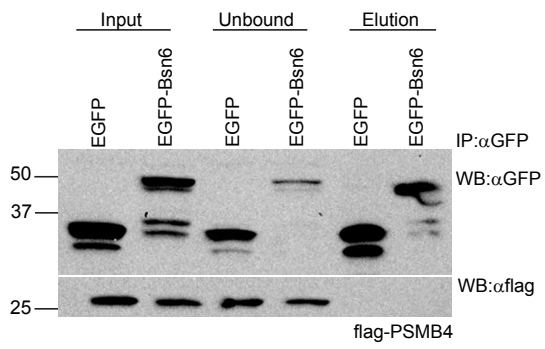

**D**

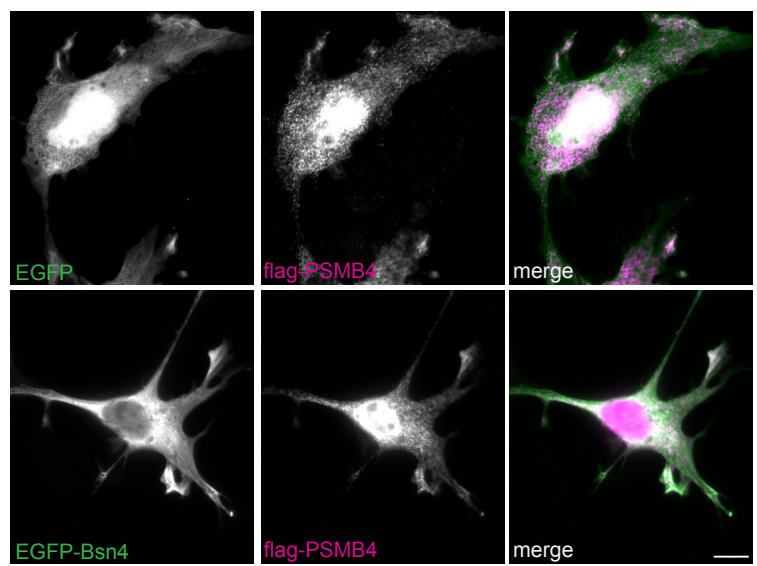

Supplement: Supplementary file 1 — Supplementary material 1 (PDF 1492 kb) [file 18_2020_3590_MOESM1_ESM.pdf]
